# Targeting body composition in an older population: do changes in movement behaviours matter? Longitudinal analyses in the PREDIMED-Plus trial

**DOI:** 10.1186/s12916-020-01847-9

**Published:** 2021-01-06

**Authors:** Aina M. Galmes-Panades, Jadwiga Konieczna, Veronica Varela-Mato, Itziar Abete, Nancy Babio, Miquel Fiol, José Antonio de Paz, Rosa Casas, Romina Olbeyra, Miguel Ruiz-Canela, Antoni Palau-Galindo, Olga Castañer, Arturo Martín-García, Ramón Estruch, Josep Vidal, Pilar Buil-Cosiales, Julia Wärnberg, Jordi Salas-Salvadó, J. Alfredo Martínez, Dora Romaguera, on behalf of the PREDIMED-Plus investigators, V. Martin, R. Pedret-Llaberia, R. Gonzalez, R. Sagarra-Álamo, F. París-Palleja, J. Balsells, J. M. Roca, T. Basora-Gallisa, J. Vizcaino, P. Llobet-Alpizarte, C. Anguera-Perpiñá, M. Llauradó-Vernet, C. Caballero, M. Garcia-Barco, M. D. Morán-Martínez, J. García-Rosselló, A. del Pozo, C. Poblet-Calaf, P. Arcelin-Zabal, X. Floresví, M. Ciutat-Benet, J. J. Cabré-Vila, F. Dolz-Andrés, M. Soler, M. Garcia-Vidal, J. Vilalta, J. Boj Casajuana, M. Ricard, F. Saiz, A. Isach, M. Sanchez-Marin Martinez, E. Granado-Font, C. Lucena-Luque, C. Mestres-Sola, N. Becerra-Tomás, G. Mestres, J. Basora, G. Mena-Sánchez, L. Barrubés-Piñol, M. Gil-Segura, N. Rosique-Esteban, S. Chig, I. Abellán-Cano, V. Ruiz-García, C. Gomez-Martinez, L. Lopez-Gonzalez, A. Salas-Huetos, I. Paz-Graniel, J. Roig-Vallverdú, C. Miñana-Garcia, L. Sánchez-Niembro, P. Hernandez-Alonso, S. Canudas, A. Díaz-López, E. Toledo, M. A. Martínez-González, Z. Vázquez, C. Razquin, M. Bes-Rastrollo, A. Gea, A. Sanchez-Tainta, B. Sanjulián-Aranguren, E. Goñi, L. Goñi, M. J. Cobo, A. Rico-Campa, F. J. Basterra-Gortari, A. Garcia-Arellano, J. Diez-Espino, O. Lecea-Juarez, J. Carlos Cenoz-Osinaga, I. Alvarez-Alvarez, M. C. Sayon-Orea, C. I. Fernandez-Lázaro, L. Ruiz-Estigarribia, J. Bartolome-Resano, A. Sola-Larraza, E. Lozano-Oloriz, B. Cano-Valles, S. Eguaras, E. Pascual Roquet-Jalmar, I. Galilea-Zabalza, H. Lancova, R. Ramallal, M. L. Garcia-Perez, V. Estremera-Urabayen, M. J. Ariz-Arnedo, C. Hijos-Larraz, C. Fernandez-Alfaro, B. Iñigo-Martinez, R. Villanueva-Moreno, S. Martin-Almendros, L. Barandiaran-Bengoetxea, C. Fuertes-Goñi, A. Lezaun-Indurain, M. J. Guruchaga-Arcelus, O. Olmedo-Cruz, L. Escriche-Erviti, R. Ansorena-Ros, R. Sanmatin-Zabaleta, J. Apalategi-Lasa, J. Villanueva-Telleria, M. M. Hernández-Espinosa, L. Herrera-Valdez, L. Dorronsoro-Dorronsoro, L. Echeverria-Lizarraga, J. A. Cabeza-Beunza, P. Fernández-Urretavizcaya, P. Gascó-García, C. Royo-Jimenez, J. Moran-Pí, F. Salazar-Fernández, F. J. Chasco-Ros, F. Cortés-Ugalde, J. J. Jurio-Burgui, P. Pascual-Pascual, A. I. Rodríguez-Ezpeleta, M. Esparza-Cáceres, C. Arroyo-Azpa, M. Rodríguez-Sanz de Galdeano, T. Forcen-Alonso, M. Armendariz-Marcotegui, A. Brugos-Larumbe, A. Arillo, B. López-Aisa, M. Moñino, A. Colom, M. Morey, M. A. Martín, E. Rayó, J. Llobera, C. Fernández-Palomeque, E. Fortuny, M. Noris, L. López, X. Rosselló, S. Munuera, F. Tomás, F. Fiol, A. Jover, J. M. Janer, C. Vallespir, I. Mattei, N. Feuerbach, M. M. Sureda, S. Vega, L. Quintana, A. Fiol, M. Amador, S. González, J. Coll, A. Moyá, T. Piqué-Sistac, M. D. Sanmartín-Fernández, M. C. Piña-Valls, M. A. Llorente San Martín, J. Pou-Bordoy, I. Cantero, C. Cristobo, I. Ibero-Baraibar, M. Zulet, J. Ágreda-Peiró, M. D. Lezáun-Burgui, N. Goñi-Ruiz, R. Bartolomé-Resano, E. Cano-Cáceres, T. Elcarte-López, E. Echarte-Osacain, B. Pérez-Sanz, I. Blanco-Platero, A. Andueza-Azcárate, A. Gimeno-Aznar, E. Ursúa-Sesma, B. Ojeda-Bilbao, J. Martinez-Jarauta, L. Ugalde-Sarasa, B. Rípodas-Echarte, M. V. Güeto-Rubio, C. Napal-Lecumberri, M. D. Martínez-Mazo, E. Arina-Vergara, A. Parra-Osés, F. Artal-Moneva, F. Bárcena-Amigo, F. Calle-Irastoza, J. Abad-Vicente, J. I. Armendáriz-Artola, P. Iñigo-Cibrian, J. Escribano-Jarauta, J. Ulibarri-delportillo, B. Churio-Beraza, Y. Monzón-Martínez, E. Madoz-Zubillaga, C. Arroniz, C. Viñas, S. Castro-Barquero, A. M. Ruiz-León, R. Losno, L. Tarés, A. Jordán, R. Soriano, M. Camafort, C. Sierra, E. Sacanella, J. M. Cots, I. Sarroca, M. García, N. Bermúdez, A. Pérez, I. Duaso, A. de la Arada, R. Hernández, C. Simón, M. A. de la Poza, I. Gil, M. Vila, C. Iglesias, N. Assens, M. Amatller, L. L. Rams, T. Benet, G. Fernández, J. Teruel, A. Azorin, M. Cubells, D. López, J. M. Llovet, M. L. Gómez, P. Climente, L. de Paula, J. Soto, C. Carbonell, C. Llor, X. Abat, A. Cama, M. Fortuny, C. Domingo, A. I. Liberal, T. Martínez, E. Yañez, M. J. Nieto, A. Pérez, E. Lloret, C. Carrazoni, A. M. Belles, C. Olmos, M. Ramentol, M. J. Capell, R. Casas, I. Giner, A. Muñoz, R. Martín, E. Moron, A. Bonillo, G. Sánchez, C. Calbó, J. Pous, M. Massip, Y. García, M. C. Massagué, R. Ibañez, J. Llaona, T. Vidal, N. Vizcay, E. Segura, C. Galindo, M. Moreno, M. Caubet, J. Altirriba, G. Fluxà, P. Toribio, E. Torrent, J. J. Anton, A. Viaplana, G. Vieytes, N. Duch, A. Pereira, M. A. Moreno, E. Sant, J. Gené, H. Calvillo, F. Pont, M. Puig, M. Casasayas, A. Garrich, E. Senar, A. Martínez, I. Boix, E. Sequeira, V. Aragunde, S. Riera, M. Salgado, M. Fuentes, E. Martín, A. Ubieto, F. Pallarés, C. Sala, A. Abilla, S. Moreno, E. Mayor, T. Colom, A. Gaspar, A. Gómez, L. Palacios, R. Garrigosa, V. Martín, S. Abajo-Olea, L. Álvarez-Álvarez, M. Rubín-García, A. Torres, P. Farias, N. Cubelos, A. Adlbi Sibai, M. Ajenjo, E. Carriedo-Ule, M. Escobar-Fernández, J. I. Ferradal-García, J. P. Fernández-Vázquez, C. González-Quintana, F. González-Rivero, M. Lavinia-Popescu, J. I. López-Gil, J. López de la Iglesia, A. Marcos-Delgado, C. Merino-Acevedo, S. Reguero-Celada, M. Rodríguez-Bul, E. Fernández-Mielgo, A. Altés, I. Vinagre, C. Mestre, J. Viaplana, M. Serra, J. Vera, T. Freitas, E. Ortega, I. Pla

**Affiliations:** 1 Consorcio CIBER, M.P. Fisiopatología de la Obesidad y la Nutrición (CIBEROBN), Instituto de Salud Carlos III (ISCIII), Madrid, Spain; 2 Research Group on Nutritional Epidemiology & Cardiovascular Physiopathology (NUTRECOR), Health Research Institute of the Balearic Islands (IdISBa), University Hospital Son Espases (HUSE), Palma de Mallorca, Spain; 3 School of Sport, Exercise and Health Science, Loughborough University, Loughborough, UK; 4 Department of Nutrition, Food Sciences and Physiology, Center for Nutrition Research, University of Navarra, Pamplona, Spain; 5 Navarra Institute for Health Research (IdiSNA), University of Navarra, Pamplona, Spain; 6 Universitat Rovira i Virgili, Departament de Bioquímica i Biotecnologia, Unitat de Nutrició Humana, Reus, Spain; 7 Institut d’Investigació Sanitària Pere Virgili (IISPV), Hospital Universitari San Joan de Reus, Reus, Spain; 8 Instituto de Biomedicina (IBIOMED), University of León, León, Spain; 9 Department of Internal Medicine, Institut d’Investigacions Biomèdiques August Pi Sunyer (IDIBAPS), Hospital Clinic, University of Barcelona, Barcelona, Spain; 10 Institut d’Investigacions Biomèdiques August Pi i Sunyer (IDIBAPS), Barcelona, Spain; 11 Department of Preventive Medicine and Public Health, IDiSNA, University of Navarra, Pamplona, Spain; 12 ABS Reus V. Centre d’Assistència Primària Marià Fortuny, SAGESSA, Reus, Spain; 13 Unit of Cardiovascular Risk and Nutrition, Institut Hospital del Mar d’Investigacions Mèdiques (IMIM), Barcelona, Spain; 14 Research Group in Gene-Environment Interactions and Health, Instituto de Biomedicina (IBIOMED), University of León, León, Spain; 15 CIBER Diabetes y Enfermedades Metabólicas (CIBERDEM), Instituto de Salud Carlos III (ISCIII), Madrid, Spain; 16 Endocrinology and Nutrition Department, Obesity Unit, Hospital Clinic, Barcelona, Spain; 17 Primary Health Care, Servicio Navarro de Salud, Navarra, Spain; 18 School of Health Sciences, University of Málaga-Institute of Biomedical Research in Malaga (IBIMA), Málaga, Spain; 19 Precision Nutrition and Cardiometabolic Health program, IMDEA Food, CEI UAM + CSIC, Madrid, Spain

**Keywords:** Physical activity, Sedentary behaviour, Body composition, Visceral adipose tissue, Isotemporal substitution

## Abstract

**Background:**

The optimal distribution between physical activity (PA) levels and sedentary behaviour (SB) for the greatest benefits for body composition among older adults with overweight/obesity and chronic health conditions remains unclear. We aimed to determine the prospective association between changes in PA and in SB with concurrent changes in body composition and to examine whether reallocating inactive time into different physical activity levels was associated with 12-month change to body composition in older adults.

**Methods:**

Longitudinal assessment nested in the PREDIMED-Plus trial. A subsample (*n* = 1564) of men and women (age 55–75 years) with overweight/obesity and metabolic syndrome from both arms of the PREDIMED-Plus trial was included in the present analysis. Participants were followed up at 6 and 12 months. Physical activity and SB were assessed using validated questionnaires. Out of 1564 participants, 388 wore an accelerometer to objectively measure inactive time and PA over a 7-day period. At each time point, participants’ body composition was measured using dual-energy X-ray absorptiometry (DXA). Standard covariate-adjusted and isotemporal substitution modelling were applied to linear mixed-effects models.

**Results:**

Increasing 30 min of total PA and moderate-to-vigorous physical activity (MVPA) was associated with significant reductions in body fat (*β* − 0.07% and − 0.08%) and visceral adipose tissue (VAT) (− 13.9 g, and − 15.6 g) at 12 months (all *p* values < 0.001). Reallocating 30 min of inactive time to MVPA was associated with reductions in body fat and VAT and with an increase in muscle mass and muscle-to-fat mass ratio (all *p* values < 0.001).

**Conclusions:**

At 12 months, increasing total PA and MVPA and reducing total SB and TV-viewing SB were associated with improved body composition in participants with overweight or obesity, and metabolic syndrome. This was also observed when substituting 30 min of inactive time with total PA, LPA and MVPA, with the greatest benefits observed with MVPA.

**Trial registration:**

International Standard Randomized Controlled Trial (ISRCTN), 89898870. Retrospectively registered on 24 July 2014

**Supplementary information:**

The online version contains supplementary material available at 10.1186/s12916-020-01847-9.

## Background

The relationship between physical activity (PA), sedentary behaviour (SB) and markers of obesity and body composition has been researched extensively in the literature [1–5]. However, a beneficial combination between SB and PA at different intensity levels for the greatest benefits for body composition among older adults with overweight and obesity remains unclear [1, 6–10]. Up-to-date, only a few studies have been conducted in older adults with chronic conditions [4, 11], and scarce research has used longitudinal or objectively measured data [8, 12–15].

Age-related changes in body composition include a decline in muscle mass and the accumulation of fat in central body regions [16], leading to physical impairment and morbidity. Greater visceral adipose tissue (VAT) seems to play a particular role in the development of chronic diseases, such as insulin resistance, type 2 diabetes (T2D) and cardiovascular disease (CVD) [12, 17, 18]. Understanding how increases in PA and reductions in SB may minimize the adverse effects of ageing on body composition would shed light as to what are the best strategies to help improve health and quality of life in older people.

Different PA dimensions (mode, frequency, duration and intensity) may affect body composition differently, depending partly on SB displacement. Isotemporal substitution models (ISMs) have been recommended as one of the most appropriate statistical analysis to explore the associations between reallocating activity patterns (time spent in PA and SB) and health outcomes, taking into account the finite concept of time (24 h/day) [19, 20]. Scarce research using ISM to explore the associations between activity patterns and body composition has been conducted in elderly cohorts, and most research had been conducted using a cross-sectional design [21, 22] or used anthropometry to assess body composition [15]. Therefore, studies using device-based longitudinal measures for activity patterns and regional body composition are warranted.

This novel study aimed to provide new evidence about the associations between SB and PA with directly quantified body composition in an ageing population with metabolic syndrome, using a longitudinal study design. The specific objectives of the present study were (a) to determine the association of concurrent changes in self-reported PA levels and SB with body composition changes measured with dual-energy X-ray absorptiometry (DXA) at 12 months follow-up and (b) to assess the impact of replacing accelerometer-derived inactive time (IT) data, as a proxy measure of SB, with PA at different intensities and with time in bed, on body composition changes.

## Methods

### Study overview and sample

The present study reports an observational prospective assessment nested in the PREDIMED-Plus study (Spain). This clinical trial aims to prevent CVD in older adults and has been described elsewhere [23, 24]. In brief, men (55–75 years) and women (60–75 years), with a body mass index (BMI) ≥ 27 and < 40 kg/m^2^ and ≥ 3 components of the metabolic syndrome (MetS) were eligible [25]. Participants were recruited into the study between 2013 and 2016 and were randomized into control or intervention arm. Members of the same households were randomized by clusters, with the couple as the unit of randomization. Participants in the intervention arm received a multicomponent weight loss intervention, based on an energy-restricted traditional Mediterranean diet (erMedDiet), PA promotion and behavioural support. Those in the control group received recommendations about an unrestricted caloric Mediterranean diet and usual care without advices on energy restriction, PA and weight loss objective [24] (the trial is still ongoing) (for further information, please visit http://www.predimedplus.com/).

**Fig. 1:**
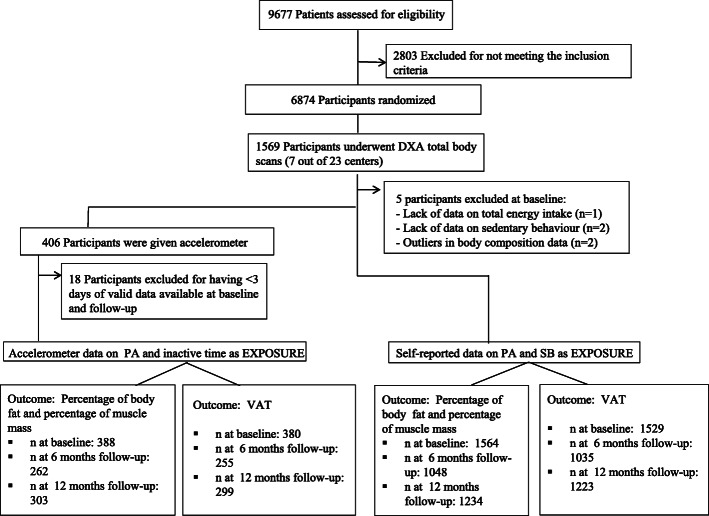
Flow chart of the study sample. *DXA* dual-energy X-ray absorptiometry, *PA* physical activity, *SB* sedentary behaviour, *VAT* visceral adipose tissue

Longitudinal data collected at baseline, 6 and 12 months from a subsample of 1564 participants from the PREDIMED-Plus trial (total sample 6874), recruited across 7 centres, of both control and intervention arms, were included in this analysis. Figure 1 shows the number of participants at each time point. All participants provided written informed consent. The study’s protocol was approved by the Research Ethics Committees from all recruiting centres according to the ethical standards of the Declaration of Helsinki. The trial was registered at the International Standard Randomized Controlled Trial (ISRCTN: http://www.isrctn.com/ISRCTN89898870). The study’s longitudinal database generated on March 25, 2019, was used for this analysis.

### Exposure assessment

#### Self-reported physical activity and sedentary behaviours

Leisure-time PA performed during a conventional month was assessed using the validated self-reported REGICOR questionnaire [26]. Time spent on SB over the last year was measured using the validated self-reported Nurses’ Health Study questionnaire [27]. Time spent in PA was calculated as a product of the frequency and duration of 6 types of activities categorized into three intensities: light PA (LPA) (< 4 Metabolic Equivalent Tasks (METs))—walking at a slow/normal pace; moderate PA (4–5.5 METs)—brisk walking and gardening; and vigorous PA (≥ 6.0 METs)—walking in the countryside, climbing stairs, exercise or playing sports [28]. Moderate-to-vigorous PA (MVPA) was calculated as the sum of moderate and vigorous PA, and total PA was determined by adding up all the activities. Time spent in total SB (counting the number of hours per day spent in a seated position) and in TV viewing was calculated as the sum of time spent in each activity during weekdays*5 and weekend-days*2. Data for these questionnaires were collected by trained interviewers in all time points. Questionnaire results are analysed in accumulated increments of 30 min/day.

#### Accelerometer measured physical activity and inactive time

Inactive time, used as a proxy measure for sedentary time, is defined as any activity that requires less than 1.5 METs during waking hours. Participants were asked to wear an accelerometer (GENEActiv, ActivInsights Ltd., Kimbolton, UK) on their non-dominant wrist continuously for 7 days. Data extracted from the GENEActiv were examined as 1-min epochs of IT (< 1.5 METs), LPA (1.5–3 METs), MVPA (> 3 METs) and time in bed (time difference between going to bed and leaving) [29–31]. Details of how the PA and IT were processed have been described [32] previously. Accelerometer measures were taken at baseline, 6- and 12-months follow-up. Accelerometer results are presented in accumulated increments of 30 min/day.

### Outcome assessment

#### Body composition

Baseline and 6 and 12 months follow-up data of total and regional body composition were taken using third-generation DXA scanners from GE Healthcare (Madison, WI, USA), using the EnCore™ software. Total body fat mass and total body muscle mass were expressed as the percentage of DXA-derived total body mass (sum of total bone, fat and muscle mass (g)). VAT was determined using the validated CoreScan software application [33]. The muscle-to-fat mass ratio was calculated dividing the total muscle mass (g) by the total fat mass (g) and multiplied by 100. DXA scans were performed by trained operators following a standard protocol and subject positioning provided by the manufacturer. DXA scanners were phantom calibrated daily according to the manufacturer’s guidelines.

### Other covariates

Baseline data for sex, age, smoking habits (categorized as never, current or former), educational level (categorized as higher education/technician, secondary education or not-completed primary education/primary education), medical conditions (T2D) and medication use (antidiabetic treatment) were self-reported. Body weight (kg) and height (m) were measured in light clothing and without shoes using a calibrated scale and a wall-mounted stadiometer, respectively. Weight and height were measured twice, and the mean value was used in the analysis. Glycated haemoglobin (HbA1c, %) was determined using standard biochemical analyses with blood samples collected after an overnight fast. Type 2 diabetes was defined as meeting any of the following criteria: self-reported diabetes at inclusion or baseline, HbA1c ≥ 6.5% or use of antidiabetic medication at baseline, such as insulin, metformin (in case of diagnosed diabetes or HbA1c ≥ 6.5%) and other medication for diabetes. A validated food frequency questionnaire [34] and the Spanish food composition tables [35] were used to estimate total energy intake (kcal/day).

### Statistical analyses

Characteristics of the study participants at baseline and at follow-up are presented as mean and standard deviations (SD) for continuous variables and absolute numbers (percentages) for categorical variables.

Main analyses were run in completers-only. Linear mixed-effects models with random intercepts at recruiting centre, family and patient levels were used to explore the associations between concurrent changes in self-reported PA (total, light and moderate-to-vigorous, in accumulated increments of 30 min/day) and SB (total and TV viewing, in accumulated increments of 30 min/day) with body composition changes (percentage of body fat, percentage of muscle mass, g of VAT) at 12 months follow-up. Changes in repeatedly measured variables were calculated as the difference between the results from each follow-up assessment (changes from 0 to 6 months follow-up and from 6 to 12 months follow-up). Firstly, minimally adjusted models were run controlling for age (years), sex, intervention arm (intervention or control group) and follow-up point (months). Multivariable-adjusted models were further adjusted for baseline variables, such as educational level, smoking status, T2D (all categorical) and height (m), as well as changes in repeatedly measured total energy intake (kcal/day), total PA (30 min/day accumulated increments) for SB exposures, and total SB (30 min/day accumulated increments) for PA exposures.

Analyses using objectively measured PA and IT in the subsample of participants providing DXA scans and accelerometer data (*n* = 388) were also performed. Linear mixed-effects models using the ISM were used to explore the impact of replacing 30 min of IT by 30 min of time in bed, LPA or MVPA on body composition changes at 12 months follow-up. These models were performed with random intercepts at the recruiting centre, family and patient levels. Prior to running the minimally- and multivariable-adjusted models (the same covariates as described above), all activity patterns at baseline and 6 and 12 months follow-up (time in bed, IT, LPA and MVPA) were divided by 30, which was considered as the unit of time equivalent to 30 min/day (according to the PA guidelines [36–38]). To account for the 24-h day finite time [20], a variable representing the total accelerometer wear time was constructed by adding up time in bed, IT, LPA and MVPA. This was entered simultaneously in all ISMs. Analyses followed the published guidelines for ISM [39]. Sensitivity analyses using the last observation carried forward (LOCF) method were used to estimate missing data at follow-up on both exposure and outcome variables. Models were repeated after excluding data measured at 6 months (due to a high number of missing data). These analyses were also performed in the subsample of participants (*n* = 388) providing accelerometer data.

Lastly, potential effect modifications by sex (men or women) were checked by adding an interaction term between sex and all exposures. Stratified analyses were conducted when a significant interaction was detected (*p* < 0.05). All analyses were conducted with the Stata v15.0. program. All *p* values < 0.05 were deemed statistically significant.

## Results

Table 1 shows the participants’ characteristics at baseline, 6 and 12 months. On average, participants at baseline were 65 years old, with a BMI of 32.5 kg/m^2^ and 48% were women. At 12 months, participants (intervention and control groups) reduced their waist circumference, BMI and total energy intake compared to baseline. At 6 and 12 months, participants accrued more total PA, LPA and MVPA and less total and TV-viewing SB compared to baseline. Reductions of percentage body fat and VAT and increased percentage of muscle mass and muscle-to-fat mass ratio were also observed at 12 months. Similar results were observed in those providing accelerometry data (see Additional file 1: Table S1).

**Table 1:** Socio-demographic, lifestyle and body composition characteristics of participants at baseline, 6 and 12 months follow-up

Parameters	Number	Baseline, mean (SD)	Number	6 months, mean (SD)	Number	12 months, mean (SD)
Socio-demographic characteristics						
Age (years)	1564	65.3 (5.0)				
Sex, women, *n* (%)	1564	751 (48)				
Type 2 diabetes, *n* (%)	1564	469 (30)				
Height (m)	1564	1.63 (0.10)				
Waist circumference (cm)	1564	107 (9.31)	1444	105 (9.52)	1404	105 (9.72)
Body mass index (kg/m^2^)	1564	32.5 (3.34)	1502	32.0 (3.49)	1496	31.8 (3.50)
Current smokers, *n* (%)	1564	197 (12.6)				
Higher education, *n* (%)	1564	333 (21.3)				
Total energy intake (kcal/day)	1564	2426 (581)	1428	2294 (467)	1400	2268 (454)
Lifestyle: physical activity and sedentary behaviour (self-reported)						
Total PA (h/day)	1564	1.22 (1.01)	1447	1.40 (1.06)	1406	1.41 (1.06)
LPA (h/day)	1564	0.47 (0.56)	1447	0.48 (0.59)	1406	0.49 (0.59)
MVPA (h/day)	1564	0.76 (0.90)	1447	0.92 (0.96)	1406	0.92 (0.97)
Total SB (h/day)	1564	5.86 (1.86)	1446	5.63 (1.77)	1406	5.51 (1.75)
TV-viewing SB (h/day)	1564	3.12 (1.64)	1446	2.91 (1.52)	1406	2.87 (1.44)
Body composition determined by DXA						
Percentage of body fat*	1564	40.5 (6.90)	1048	39.7 (7.06)	1234	39.6 (7.04)
VAT (kg)	1529	2.30 (0.89)	1035	2.17 (0.86)	1223	2.21 (0.88)
Percentage of muscle mass*	1564	56.5 (6.56)	1048	57.2 (6.70)	1234	57.2 (6.68)
Muscle-to-fat mass ratio**	1564	147 (44.1)	1048	152 (48.3)	1234	153 (48.1)

Values are mean (SD) for continuous variables, and *n* (percentage) for categorical variables

*DXA* dual-energy X-ray absorptiometry, *VAT* visceral adipose tissue, *PA* physical activity, *LPA* light physical activity, *MVPA* moderate-to-vigorous physical activity, *SB* sedentary behaviour

*Percentage of body fat and percentage of muscle mass were calculated taking into account muscle mass, fat mass and bone mass measured with a whole-body DXA scan

**Muscle-to-fat mass ratio was calculated as (total muscle mass in g/total fat mass in g) × 100

Table 2 shows the *β* coefficients (95% CIs) for the associations between concurrent changes in self-reported leisure-time PA, self-reported SB (both per 30-min accumulated increments) and body composition. After adjustment for potential confounders, increasing 30 min of total PA was significantly associated with a decrease in body fat (*β* − 0.07%, 95% CIs − 0.10; − 0.04%) and VAT (− 13.9 g; − 21.5; − 6.23) and increased muscle mass (0.07%; 0.04; 0.10) and muscle-to-fat mass ratio (0.41; 0.15; 0.67). Increases of 30 min of MVPA were linked to significantly reduced body fat (− 0.08%, − 0.11; − 0.04%) and VAT (− 15.6 g; − 24.1; − 7.25) and with increased muscle mass (0.07%; 0.04; 0.10) and muscle-to-fat mass ratio (0.44; 0.15; 0.72). Overall, 30 more minutes of total and TV-viewing SB were associated with significantly greater body fat and lower muscle mass. No significant associations were observed for LPA.

**Table 2:** Association of concurrent changes in self-reported leisure-time physical activity and sedentary behaviour in accumulated increments of 30 min with body composition: analyses in completers-only

	Percentage of body fat		VAT (g)		Percentage of muscle mass		Muscle-to-fat mass ratio	
	*β* (95% CI)	*p* value	*β* (95% CI)	*p* value	*β* (95% CI)	*p* value	*β* (95% CI)	*p* value
**Physical activity**								
**Total PA**								
Minimally adjusted	− 0.08 (− 0.11; − 0.05)	< 0.001	− 15.1 (− 22.7; − 7.49)	< 0.001	0.08 (0.05; 0.11)	< 0.001	0.46 (0.20; 0.71)	0.001
Multivariable-adjusted	− 0.07 (− 0.10; − 0.04)	< 0.001	− 13.9 (− 21.5; − 6.23)	< 0.001	0.07 (0.04; 0.10)	< 0.001	0.41 (0.15; 0.67)	0.002
**LPA**								
Minimally-adjusted	− 0.02 (− 0.07; 0.02)	0.298	− 1.69 (− 13.2; 9.87)	0.775	0.02 (− 0.02; 0.07)	0.311	0.10 (− 0.29; 0.49)	0.629
Multivariable-adjusted	− 0.03 (− 0.07; 0.02)	0.264	− 2.23 (− 13.8;9.36)	0.706	0.02 (− 0.02; 0.07)	0.283	0.11 (− 0.28; 0.50)	0.585
**MVPA**								
Minimally-adjusted	− 0.09 (− 0.12; − 0.06)	< 0.001	− 17.4 (− 25.8; − 9.05)	< 0.001	0.08 (0.05; 0.12)	< 0.001	0.50 (0.22; 0.78)	0.001
Multivariable-adjusted	− 0.08 (− 0.11; − 0.04)	< 0.001	− 15.6 (− 24.1; − 7.25)	< 0.001	0.07 (0.04; 0.10)	< 0.001	0.44 (0.15; 0.72)	0.003
**Sedentary behaviour**								
**Total SB**								
Minimally-adjusted	0.04 (0.02; 0.06)	< 0.001	5.46 (0.78; 10.1)	0.022	− 0.04 (− 0.06; − 0.02)	< 0.001	− 0.17 (− 0.32; − 0.01)	0.040
Multivariable-adjusted	0.03 (0.01; 0.05)	< 0.001	4.37 (− 0.35; 9.08)	0.070	− 0.03 (− 0.05; − 0.01)	< 0.001	− 0.14 (− 0.30; 0.02)	0.097
**TV-viewing SB**								
Minimally-adjusted	0.03 (0.01; 0.05)	0.007	0.56 (− 5.17; 6.29)	0.848	− 0.03 (− 0.05; − 0.01)	0.007	− 0.10 (− 0.29; 0.09)	0.305
Multivariable-adjusted	0.03 (0.01; 0.05)	0.014	− 0.01 (− 5.74; 5.72)	0.996	− 0.03 (− 0.05; − 0.01)	0.012	− 0.08 (− 0.28; 0.11)	0.390

The values shows the *β* coefficients (95% CIs). These represent the change in outcome variables (percentage of body fat, VAT (g), percentage of muscle mass and muscle-to-fat mass ratio), when increasing 30 min/day of each exposure variable (total PA, LPA, MVPA, total SB and TV-viewing SB). Mixed-effects linear models with random intercepts at recruiting centre, family and patient level were used. Analyses included only completers. Minimally-adjusted model: age, sex, intervention arm and time. The multivariable-adjusted model was further adjusted for baseline variables, such as educational level, smoking, diabetes, height, as well as repeatedly measured total energy intake, total PA (for SB exposures), and total SB (for PA exposures). The *n* of each outcome at baseline was for the percentage of body fat *n* = 1564, for VAT *n* = 1529, for the percentage of muscle mass *n* = 1564 and for muscle-to-fat mass ratio *n* = 1564; at 6 months was for the percentage of body fat *n* = 1048, for VAT *n* = 1035, for the percentage of muscle mass *n* = 1048 and for muscle-to-fat mass ratio *n* = 1048; at 12 months was for the percentage of body fat *n* = 1234, for VAT *n* = 1223, for the percentage of muscle mass *n* = 1234 and for muscle-to-fat mass ratio *n* = 1234

*VAT* visceral adipose tissue, *PA* physical activity, *LPA* light physical activity, *MVPA* moderate-to-vigorous physical activity, *SB* sedentary behaviour

**Fig. 2:**
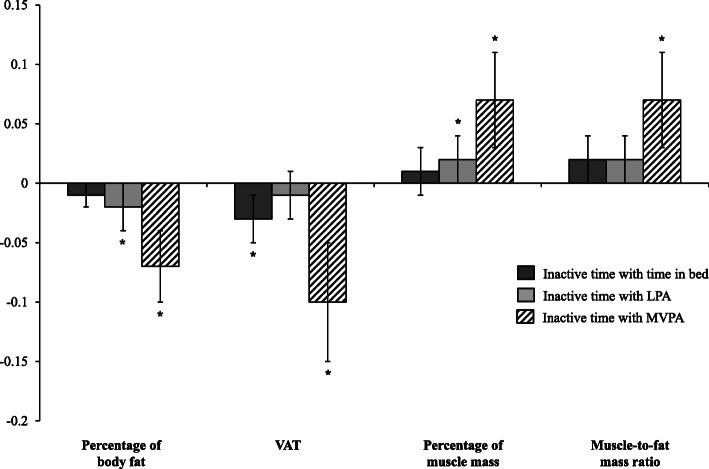
Isotemporal substitution of inactive time (30 min/day) with time in bed and physical activity on standardized body composition (*z*-score): analyses in completers only. The values shown are *β* (95% CI). These represent the change in outcome variables (*z*-scores) when substituting 30 min/day of inactive time with time in bed and physical activity. Mixed-effects linear models with random intercepts at recruiting centre, family and patient level were used to assess the isotemporal substitution of inactive time with time in bed, LPA and MVPA, adjusting for age, sex, intervention arm, time, educational level, smoking, diabetes, height, repeatedly measured total energy intake and total wear time. LPA, light physical activity; MVPA, moderate-to-vigorous physical activity; VAT, visceral adipose tissue. *p value < 0.05

Table 3 shows the *β* coefficients (95% CIs) for the ISM; Fig. 2 shows the ISM with the changes in body composition standardized as *z*-scores to aid comparability. After adjusting for potential confounders, reallocating 30 min/day of IT with time in bed, LPA and MVPA was associated with lower VAT (*β* − 23.8 g, − 11.2 g and − 92.4 g) and body fat (*β* − 0.09%, − 0.13% and − 0.54%) and with an increased muscle mass (*β* 0.08%, 0.12% and 0.51%) and muscle-to-fat mass ratio (*β* 0.89, 0.90 and 3.74), with the strongest associations seen in MVPA.

**Table 3:** Isotemporal substitution of inactive time (30 min/day) with time in bed and physical activity measured with an accelerometer in body composition changes: longitudinal analyses in completers-only

	Percentage of body fat		VAT (g)		Percentage of muscle mass		Muscle-to-fat mass ratio	
	*β* (95% CI)	*p* value	*β* (95% CI)	*p* value	*β* (95% CI)	*p* value	*β* (95% CI)	*p* value
**Inactive time with time in bed**								
Minimally-adjusted	− 0.10 (− 0.20; 0.00)	0.051	− 26.9 (− 46.5; − 7.27)	0.007	0.09 (− 0.01; 0.19)	0.067	0.93 (0.03; 1.83)	0.042
Multivariable-adjusted	− 0.09 (− 0.19; 0.01)	0.079	− 23.8 (− 43.3; − 4.27)	0.017	0.08 (− 0.02; 0.18)	0.097	0.89 (− 0.01; 1.79)	0.052
**Inactive time with LPA**								
Minimally-adjusted	− 0.13 (− 0.25; − 0.00)	0.044	− 11.8 (− 36.1; 12.6)	0.343	0.12 (0.00; 0.24)	0.046	0.87 (− 0.24; 1.97)	0.124
Multivariable-adjusted	− 0.13 (− 0.25; − 0.00)	0.042	− 11.2 (− 35.4; 13.0)	0.363	0.12 (0.00; 0.25)	0.042	0.90 (− 0.20; 2.01)	0.110
**Inactive time with MVPA**								
Minimally-adjusted	− 0.53 (− 0.76; − 0.29)	< 0.001	− 98.7 (− 146; − 52.0)	< 0.001	0.50 (0.27; 0.73)	< 0.001	3.51 (1.43; 5.60)	0.001
Multivariable-adjusted	− 0.54 (− 0.78; − 0.31)	< 0.001	− 92.4 (− 139; − 45.6)	< 0.001	0.51 (0.28; 0.74)	< 0.001	3.74 (1.64; 5.85)	< 0.001

The values show the *β* coefficients (95% CIs). These represent the change in outcome variables when substituting 30 min/day of inactive time with time in bed and physical activity. Isotemporal mixed-effects linear models with random intercepts at recruiting centre, family and patient level were used. Analyses included only completers. Minimally-adjusted model: age, sex, intervention arm, time and total wear time. The multivariable-adjusted model was further adjusted for baseline variables, such as educational level, smoking, diabetes, height, and repeatedly measured total energy intake. The *n* of each outcome at baseline was for the percentage of body fat *n* = 388, for VAT *n* = 380, for the percentage of muscle mass *n* = 388 and for muscle-to-fat mass ratio *n* = 388; at 6 months was for the percentage of body fat *n* = 262, for VAT *n* = 255, for the percentage of muscle mass *n* = 262 and for muscle-to-fat mass ratio *n* = 262; at 12 months was for the percentage of body fat *n* = 303, for VAT *n* = 299, for the percentage of muscle mass *n* = 303 and for muscle-to-fat mass ratio *n* = 303

*VAT* visceral adipose tissue, *LPA* light physical activity, *MVPA* moderate-vigorous physical activity

No major differences were observed when repeating the models in the whole sample after replacing missing data using the LOCF method (see Additional file 1: Table S2). No significant differences were found when repeating ISM in the subsample after replacing missing data using the LOCF method (see Additional file 1: Table S3). No major differences were found when linear mixed-effects models were performed in the subsample providing accelerometer data (*n* = 388) (see Additional file 1: Table S4). No modification effect by sex was observed. The results were consistent after repeating the models with completers only, excluding the 6 months’ data.

## Discussion

Results from this longitudinal study suggest that increasing total PA and MVPA were associated with an improved body composition phenotype in a sample of older adults with overweight or obesity and the MetS. Greater total SB and, to a lesser extent, TV-viewing sedentary time were associated with a worsen body composition. Overall, this study highlights that replacing 30 min a day of IT with an equal amount of MVPA, LPA and time in bed resulted in significantly improved markers of body composition.

These findings are consistent with previous cross-sectional research in adult populations [3, 32, 40], which have found a hazardous relationship between SB and markers of body composition, including body fat, VAT and muscle mass [3, 9, 32, 40, 41]. The present results showed that greater SB is associated with greater body fat and lower muscle mass in an ageing population, resulting in greater cardiometabolic risk and disability. In line with our findings, other authors found that increasing total PA and MVPA improve body composition [3, 8, 40] and reduce the accumulation of VAT [8, 10, 42, 43], yet no effects associated to LPA and body composition have been reported with the present results based on self-reported data.

Limited research using the ISM in older adults is available, and only isolated reports in general adult populations with chronic conditions, such as the MetS [44, 45], or using data from DXA scans are available [32]. However, no research using the same methods as this study in older adults has been found, limiting the opportunities for comparison. The use of ISM makes it possible to analyse the effect of substituting one behaviour for another. This type of analysis allows a better understanding of the association between PA and sedentarism, and health. It takes into account aspects of daily life, such as the need to replace one behaviour with another due to limited time (24 h/day). This is an important advantage to take into account, compared to other statistical analyses. Cross-sectional research conducted in adults (≥ 18 years) [46, 47] showed similar beneficial effects of replacing a unit of time spent inactive with equal amounts of LPA, MVPA or sleep in body composition markers using anthropometric measures. However, if this relationship persists over time remains unclear.

Our results showed that replacing IT with LPA is associated with improved body composition changes (body fat and muscle mass). However, the greatest changes in body composition were observed replacing IT with MVPA, with significant changes in all outcome variables (percentage of body fat, VAT, percentage of muscle mass and muscle-to-fat mass ratio, *p* value < 0.001). Similar results have been observed in previous cross-sectional research in adults [32] and in longitudinal studies performed with children [48, 49]. Therefore, the present results build on previous knowledge in other populations and indicate that replacing IT with any other activity behaviour has a beneficial impact on body composition in older adults with an incremental effect according to the intensity level. Indeed, replacing 30 min/day of IT with equal amounts of time in bed, LPA and MVPA was associated with a decrease in body fat of − 0.09%, − 0.13% and − 0.54%, respectively. Therefore, these results showed the close interactions between IT, PA and health and highlight the need for them to be treated jointly. These research also highlights that to promote the greater body composition changes, MVPA is the most effective form of PA [8, 17, 32, 40, 43, 50], yet increasing LPA in older adults with chronic conditions would also be of benefit for an improved health profile [13, 40, 47, 51–54]. Overall, small beneficial changes in body composition were observed when replacing IT with time in bed, which is similar to previous research [32, 52]. Previous research has shown a link between sleep and obesity [55]; nevertheless, given the potential measurement errors in the measurement of sleep in our study, further research using gold standard measures to assess sleep in older adults is needed.

Marked strengths of this study were the use of a longitudinal design in a large cohort of older men and women, with overweight/obesity and MetS across different communities in Spain using objective measurements. However, this study involved a homogeneous sample of Caucasian men and women within narrow ranges of BMI, age and with worsen metabolic health profile, limiting the opportunities for extrapolation into other ethnicities and with healthier individuals. Therefore, it is recommended for future research to be replicated in different ethnic groups with different lifestyles and fat distribution. It is important to highlight the novelty of the present study, with repeated measures of body composition using gold standard methods, such as DXA [56, 57], and the measurement of exposure variables with validated questionnaires and with accelerometer data in a subsample. Several complex and sophisticated statistical analyses were performed to assess our results. Some limitation to highlight is the use of questionnaires to obtain data on PA and SB within the larger sample. Although these were validated methods and have facilitated access to a larger sample size, we need to take into account that questionnaires might be subjected to potential reporting biases. It is important to mention that the GENEactiv is not able to differentiate between sitting and standing position or to differentiate time in bed from sleeping [29–31]; thus, further similar research using other monitors capable to differentiate between these behaviours is recommended. Finally, there was a considerable loss of data from the DXA scan at 6 and 12 months’ visits. Nevertheless, results were mostly consistent when imputing missing data in those subjects using the LOCF method.

## Conclusions

Results from this longitudinal study indicate that increases in PA and reductions of SB over 12 months follow-up were associated with an improved body composition profile in older adults with overweight or obesity and MetS. Replacing IT with any PA and time in bed were associated with improvements in body composition. Based on the present results, the promotion of MVPA would provide the greatest health benefits in older adults, followed by LPA. Taking this into account, interventions promoting LPA might be more appealing in terms of feasibility and sustainability, as it will help increase attrition rates and reduce participant and delivery burden as they will not need continuous supervision, making them a low-cost and easy option to be implemented at home or care homes. Future intervention trials are needed to confirm causality of the effect of PA and SB on body composition changes in older adults with chronic conditions.

## Supplementary Information

**Additional file 1:** **Table S1.** Socio-demographic, lifestyle and body composition characteristics of participants at baseline, 6 and 12 months follow-up in a subsample of participants with accelerometer data available. **Table S2.** Association of concurrent changes in self-reported leisure time physical activity and sedentary behaviour in accumulated increments of 30 minutes with body composition: longitudinal analysis with last observation carried forward method. **Table S3.** Isotemporal substitution of inactive time (30 min/day) with time in bed and physical activity measured with accelerometer in body composition changes: longitudinal analyses with last observation carried forward method. **Table S4.** Association of concurrent changes in self-reported leisure time physical activity and sedentary behaviour in accumulated increments of 30 minutes with body composition in a subsample of 388 participants: analyses in completers-only.

**Additional file 2.** List of PREDIMED-Plus study investigators.

## Data Availability

There are restrictions on the availability of data for the PREDIMED-Plus trial, due to the signed consent agreements around data sharing, which only allow access to external researchers for studies following the project purposes. Requestors wishing to access the PREDIMED-Plus trial data used in this study can make a request to the PREDIMED-Plus trial Steering Committee chair: jordi.salas@urv.cat. The request will then be passed to members of the PREDIMED-Plus Steering Committee for deliberation.

## Abbreviations

BMI: Body mass index
CVD: Cardiovascular disease
DXA: Dual-energy X-ray absorptiometry
erMedDiet: Energy-restricted traditional Mediterranean diet
HbA1c: Glycated haemoglobin
ISM: Isotemporal substitution model
IT: Inactive time
LOCF: Last observation carried forward
LPA: Light physical activity
MET: Metabolic equivalent task
MetS: Metabolic syndrome
MVPA: Moderate-to-vigorous physical activity
PA: Physical activity
SB: Sedentary behaviour
SD: Standard deviation
T2D: Type 2 diabetes
VAT: Visceral adipose tissue
