# Supplementary material for: Targeting body composition in an older population: do changes in movement behaviours matter? Longitudinal analyses in the PREDIMED-Plus trial
Source: BMC Med. 2021 Jan 6;19:3. doi: 10.1186/s12916-020-01847-9 (PMC7786490; doi:10.1186/s12916-020-01847-9)
Supplement: Supplementary file 1 — Additional file 1: Table S1. Socio-demographic, lifestyle and body composition characteristics of participants at baseline, 6 and 12 months follow-up in a subsample of participants with accelerometer data available. Table S2. Association of concurrent changes in self-reported leisure time physical activity and sedentary behaviour in accumulated increments of 30 minutes with body composition: longitudinal analysis with last observation carried forward method. Table S3. Isotemporal substitution of inactive time (30 min/day) with time in bed and physical activity measured with accelerometer in body composition changes: longitudinal analyses with last observation carried forward method. Table S4. Association of concurrent changes in self-reported leisure time physical activity and sedentary behaviour in accumulated increments of 30 minutes with body composition in a subsample of 388 participants: analyses in completers-only. [file 12916_2020_1847_MOESM1_ESM.docx]

| **Supplementary Table 1.** Socio-demographic , lifestyle and body composition characteristics of participants at baseline, 6 and 12 months follow-up in a subsample of participants with accelerometer data available | | | | | | | | |
| --- | --- | --- | --- | --- | --- | --- | --- | --- |
| Parameters | n | Baseline  Mean (SD) | n | 6 months  Mean (SD) | | n | | 12 months  Mean (SD) |
| Socio-demographic characteristics | | | | | | | | |
| Age (years) | 388 | 65.4 (4.98) |  | |  | |  |  |
| Sex, Women, n (%) | 388 | 178 (45.9) |  | |  | |  |  |
| Type 2 Diabetes, n (%) | 388 | 119 (30.7) |  | |  | |  |  |
| Height (m) | 388 | 1.63 (0.09) |  | |  | |  |  |
| Waist circumference (cm) | 388 | 107 (9.59) | 355 | | 104 (9.82) | | 344 | 104 (9.93) |
| Body mass index (kg/m^2^) | 388 | 32.7 (3.39) | 369 | | 31.9 (3.54) | | 367 | 31.9 (3.53) |
| Current smokers, n (%) | 388 | 41 (10.6) |  | |  | |  |  |
| Higher education, n (%) | 388 | 73 (18.8) |  | |  | |  |  |
| Total energy intake (kcal/day) | 388 | 2436 (589) | 353 | | 2247 (431) | | 344 | 2257 (476) |
| Lifestyle: physical activity, inactive time and time in bed measure with accelerometer | | | | | | | | |
| Time in bed (h/day) | 388 | 8.08 (1.24) | 266 | | 8.06 (1.36) | | 262 | 8.09 (1.27) |
| LPA (h/day) | 388 | 2.62 (1.07) | 266 | | 2.75 (1.16) | | 262 | 2.71 (1.18) |
| MVPA (h/day) | 388 | 0.73 (0.56) | 266 | | 0.79 (0.64) | | 262 | 0.76 (0.60) |
| Inactive time (h/day) | 388 | 8.22 (2.05) | 266 | | 8.09 (1.98) | | 262 | 8.18 (2.18) |
| Body composition determined by DXA | | | | | | | | |
| Percentage of body fat* | 388 | 40.2 (7.02) | 262 | | 38.9 (7.38) | | 303 | 39.1 (7.35) |
| VAT (kg) | 380 | 2.27 (0.91) | 255 | | 2.07 (0.83) | | 299 | 2.14 (0.93) |
| Percentage of muscle mass* | 388 | 56.7 (6.66) | 262 | | 57.9 (6.99) | | 303 | 57.7 (6.96) |
| Muscle-to-fat mass ratio** | 388 | 149 (45.9) | 262 | | 159 (54.1) | | 303 | 157 (52.8) |

Values are mean (SD) for continuous variables, and n (percentage) for categorical variables. Abbreviation: DXA; dual-energy X-ray absorptiometry, VAT; visceral adipose tissue, LPA; light physical activity, MVPA; moderate-to-vigorous physical activity, SB; sedentary behaviour.*Percentage of body fat and percentage of muscle mass were calculated taking into account muscle mass, fat mass and bone mass measured with a whole body DXA scan. **muscle-to-fat mass ratio was calculated (total muscle mass in g / total fat mass in g)*100.

| **Supplementary Material Table 2.** Association of concurrent changes in self-reported leisure time physical activity and sedentary behaviour in accumulated increments of 30 minutes with body composition: Longitudinal analysis with last observation carried forward method. | | | | | | | | | |
| --- | --- | --- | --- | --- | --- | --- | --- | --- | --- |
|  | Percentage of body fat | | VAT (g) | | Percentage of muscle mass | | Muscle-to-fat mass ratio | | |
|  | *β (*95% CI) | *p-value* | *β (*95% CI) | *p-value* | *β (*95% CI) | *p-value* | *β (*95% CI) | *p-value* | |
| **Physical activity** | | | | | | | | | |
| **Total PA** |  |  |  |  |  |  |  | |  |
| Minimally-adjusted | -0.06 (-0.09;-0.04) | <0.001 | -10.8 (-16.8;-4.83) | <0.001 | 0.06 (0.03;0.08) | <0.001 | 0.40 (0.18;0.61) | | <0.001 |
| Multivariable-adjusted | -0.06 (-0.08;-0.03) | <0.001 | -9.61 (-15.6;-3.61) | 0.002 | 0.05 (0.03;0.08) | <0.001 | 0.36 (0.14;0.58) | | 0.001 |
| **LPA** |  |  |  |  |  |  |  | |  |
| Minimally-adjusted | 0.00 (-0.04;0.04) | 0.995 | 4.95 (-3.78;13.7) | 0.267 | 0.00 (-0.04;0.04) | 0.994 | 0.01 (-0.31;0.33) | | 0.935 |
| Multivariable-adjusted | 0.00 (-0.04;0.04) | 0.955 | 4.96 (-3.75;13.7) | 0.264 | -0.00 (-0.04;0.04) | 0.965 | 0.01 (-0.31;0.33) | | 0.963 |
| **MVPA** |  |  |  |  |  |  |  | |  |
| Minimally-adjusted | -0.08 (-0.11;-0.05) | <0.001 | -16.4 (-23.1;-9.75) | <0.001 | 0.07 (0.05;0.10) | <0.001 | 0.48 (0.24;0.73) | | <0.001 |
| Multivariable-adjusted | -0.07 (-0.10;-0.04) | <0.001 | -15.0 (-21.7;-8.27) | <0.001 | 0.07 (0.04;0.09) | <0.001 | 0.44 (0.20;0.69) | | <0.001 |
| **Sedentary Behaviour** | | | | | | | | | |
| **Total SB** |  |  |  |  |  |  |  | |  |
| Minimally-adjusted | 0.03 (0.02;0.05) | <0.001 | 4.84 (1.27;8.42) | 0.008 | -0.03 (-0.04;-0.01) | <0.001 | -0.16 (-0.29;-0.03) | | 0.016 |
| Multivariable-adjusted | 0.03 (0.01;0.04) | 0.001 | 3.79 (0.21;7.38) | 0.038 | -0.02 (-0.04;-0.01) | 0.001 | -0.12 (-0.25;0.01) | | 0.062 |
| **TV-viewing SB** |  |  |  |  |  |  |  | |  |
| Minimally-adjusted | 0.02 (0.00;0.04) | 0.033 | 1.11 (-3.39;5.60) | 0.630 | -0.02 (-0.04;-0.00) | 0.030 | -0.07 (-0.24;0.09) | | 0.382 |
| Multivariable-adjusted | 0.02 (-0.00;0.04) | 0.066 | 0.38 (-4.10;4.87) | 0.867 | -0.02 (-0.04;0.00) | 0.058 | -0.05 (-0.22;0.11) | | 0.511 |

Values shows the β-coefficients (95% CIs). These represent the change in outcome variables (percentage of body fat, VAT (g), percentage of muscle mass and muscle-to-fat mass ratio), when increase 30 min/day each exposure variable (total PA, LPA, MVPA, total SB and TV-viewing SB). Mixed-effects linear models with random intercepts at recruiting center, family and patient level were used after replacing missing data using LOCF method. Minimally-adjusted model was adjusted for age, sex, intervention arm, follow-up time. Multivariable-adjusted model was further adjusted for baseline variables, such as educational level, smoking, diabetes, height, as well as repeatedly measured total energy intake, physical activity (30 min/day accumulated increments) (models with SB as an exposure) and sedentary behavior (30 min/day accumulated increments) (models with PA as an exposure). Abbreviation: VAT; visceral adipose tissue, PA; physical activity, LPA; light physical activity, MVPA; moderate-to-vigorous physical activity, SB; sedentary behaviour. The n of each outcome at baseline was: for percentage of body fat n= 1564, for VAT n=1529, for percentage of muscle mass n=1564, and for muscle-to-fat mass ratio n=1564.

| **Supplementary Material Table 3.** Isotemporal substitution of inactive time (30 min/day) with time in bed and physical activity measured with accelerometer in body composition changes: Longitudinal analyses with last observation carried forward method. | | | | | | | | | |
| --- | --- | --- | --- | --- | --- | --- | --- | --- | --- |
|  | Percentage of body fat | | VAT (g) | | Percentage of muscle mass | | Muscle-to-fat mass ratio | | |
|  | *β (*95% CI) | *p-value* | *β (*95% CI) | *p-value* | *β (*95% CI) | *p-value* | *β (*95% CI) | *p-value* | |
| **Inactive time 🡪 Time in bed** |  |  |  |  |  |  |  | |  |
| Minimally-adjusted | -0.10 (-0.19;-0.02) | 0.017 | -18.3 (-34.3;-2.29) | 0.025 | 0.10 (0.01;0.18) | 0.021 | 0.94 (0.18;1.71) | | 0.016 |
| Multivariable-adjusted | -0.09 (-0.17;-0.01) | 0.035 | -15.6 (-31.5;-0.37) | 0.056 | 0.09 (0.00;0.17) | 0.040 | 0.87 (0.10;1.63) | | 0.027 |
| **Inactive time 🡪 LPA** |  |  |  |  |  |  |  | |  |
| Minimally-adjusted | -0.13 (-0.22;-0.03) | 0.012 | -8.33 (-27.3;10.7) | 0.390 | 0.12 (0.03;0.22) | 0.012 | 0.78 (-0.13;1.69) | | 0.091 |
| Multivariable-adjusted | -0.12 (-0.22;-0.03) | 0.013 | -7.40 (-26.3;11.5) | 0.443 | 0.12 (0.03;0.22) | 0.013 | 0.80 (-0.11;1.71) | | 0.085 |
| **Inactive time 🡪 MVPA** |  |  |  |  |  |  |  | |  |
| Minimally-adjusted | -0.41 (-0.60;-0.22) | <0.001 | -71.1 (-108;-34.7) | <0.001 | 0.39 (0.20;0.58) | <0.001 | 2.79 (1.03;4.55) | | 0.002 |
| Multivariable-adjusted | -0.42 (-0.61;-0.23) | <0.001 | -67.6 (-104;-31.3) | <0.001 | 0.40 (0.21;0.59) | <0.001 | 2.94 (1.18;4.71) | | 0.001 |

Values shows the β-coefficients (95% CIs). These represent the change in outcome variables when substituting 30 min/day of inactive time with time in bed and physical activity. Isotemporal mixed-effect linear models with random intercepts at recruiting center, family and patient level were used after replacing missing data using LOCF method. Minimally-adjusted model: age, sex, intervention arm, follow-up time and total wear time. Multivariable-adjusted model was further adjusted for baseline variables, such as educational level, smoking, diabetes, height, as well as repeatedly measured total energy intake. Abbreviation: VAT; visceral adipose tissue, LPA; light physical activity, MVPA; moderate-vigorous physical activity. The n of each outcome at baseline was: for percentage of body fat n= 388, for VAT n=380, for percentage of muscle mass n=388, and for muscle-to-fat mass ratio n=388.

| **Supplementary Material Table 4.** Association of concurrent changes in self-reported leisure time physical activity and sedentary behaviour in accumulated increments of 30 minutes with body composition in a subsample of 388 participants: analyses in completers-only. | | | | | | | | | | | |
| --- | --- | --- | --- | --- | --- | --- | --- | --- | --- | --- | --- |
|  | Total body fat (%) | | VAT (g) | | | Total muscle mass (%) | | | Ratio muscle to fat mass | | |
|  | *β (*95% CI) | *p-value* | *β (*95% CI) | *p-value* | | *β (*95% CI) | *p-value* | | *β (*95% CI) | | *p-value* |
| **Physical activity** | | | | | | | | | | | |
| **Total PA** |  |  |  |  |  | | |  |  | |  |
| Minimally-adjusted | -0.14 (-0.22;-0.05) | 0.002 | -11.7 (-28.4;4.94) | 0.168 | 0.13 (0.04;0.21) | | | 0.003 | 1.10 (0.30;1.90) | | 0.007 |
| Multivariable-adjusted | -0.13 (-0.21;-0.04) | 0.004 | -9.78 (-26.4;6.82) | 0.248 | 0.12 (0.04;0.20) | | | 0.005 | 1.03 (0.23;1.84) | | 0.012 |
| **LPA** |  |  |  |  |  | | |  |  | |  |
| Minimally-adjusted | -0.07 (-0.21;0.07) | 0.338 | -10.2 (-38.1;17.7) | 0.474 | 0.07 (-0.07;0.21) | | | 0.355 | 0.75 (-0.60;2.11) | | 0.275 |
| Multivariable-adjusted | -0.06 (-0.21;0.08) | 0.376 | -9.65 (-37.3;18.0) | 0.495 | 0.06 (-0.08;0.20) | | | 0.397 | 0.72 (-0.64;2.07) | | 0.300 |
| **MVPA** |  |  |  |  |  | | |  |  | |  |
| Minimally-adjusted | -0.15 (-0.24;-0.05) | 0.004 | -10.4 (-29.4;8.51) | 0.280 | 0.14 (0.04;0.23) | | | 0.005 | 1.08 (0.17;1.99) | | 0.020 |
| Multivariable-adjusted | -0.14 (-0.23;-0.04) | 0.007 | -8.12 (-26.9;10.7) | 0.398 | 0.13 (0.03;0.22) | | | 0.010 | 1.01 (0.10;1.92) | | 0.030 |
| **Sedentary Behaviour** | | | | | | | | | | | |
| **Total SB** |  |  |  |  |  | | |  |  |  | |
| Minimally-adjusted | 0.06 (0.00;0.12) | 0.034 | 3.65 (-7.12;14.4) | 0.507 | -0.06 (-0.11;-0.00) | | | **0.038** | -0.31 (-0.83;0.20) | 0.234 | |
| Multivariable-adjusted | 0.05 (-0.00;0.11) | 0.063 | 1.86 (-8.85;12.6) | 0.733 | -0.05 (-0.10;0.00) | | | 0.068 | -0.25 (-0.77;0.26) | 0.334 | |
| **TV-viewing SB** |  |  |  |  |  | | |  |  |  | |
| Minimally-adjusted | 0.05 (-0.02;0.12) | 0.150 | -2.03 (-15.1;11.1) | 0.761 | -0.05 (-0.12;0.01) | | | 0.128 | -0.33 (-0.95;0.29) | 0.293 | |
| Multivariable-adjusted | 0.05 (-0.02;0.11) | 0.183 | -1.78 (-14.9;11.4) | 0.791 | -0.05 (-0.11;0.02) | | | 0.157 | -0.31 (-0.93;0.32) | 0.339 | |

Values shows the β-coefficients (95% CIs). These represent the change in outcome variables (percentage of body fat, VAT (g), percentage of muscle mass and muscle-to-fat mass ratio), when increase 30 min/day each exposure variable (total PA, LPA, MVPA, total SB and TV-viewing SB). Mixed-effects linear models with random intercepts at recruiting center, family and patient level were used in completers only. Minimally-adjusted model was adjusted for age, sex, intervention arm, follow-up time. Multivariable-adjusted model was further adjusted for baseline variables, such as educational level, smoking, diabetes, height, as well as repeatedly measured total energy intake, physical activity (30 min/day accumulated increments) (models with SB as an exposure) and sedentary behavior (30 min/day accumulated increments) (models with PA as an exposure). Abbreviation: VAT; visceral adipose tissue, PA; physical activity, LPA; light physical activity, MVPA; moderate-to-vigorous physical activity, SB; sedentary behaviour. The n of each outcome at baseline was: for total body fat n= 388, for VAT n=380, for total muscle mass n=388, and for muscle mass/fat mass n=388.
