# Supplementary material for: Targeting body composition in an older population: do changes in movement behaviours matter? Longitudinal analyses in the PREDIMED-Plus trial
Source: BMC Med. 2021 Jan 6;19:3. doi: 10.1186/s12916-020-01847-9 (PMC7786490; doi:10.1186/s12916-020-01847-9)
Supplement: Supplementary file 2 — Additional file 2. List of PREDIMED-Plus study investigators. [file 12916_2020_1847_MOESM2_ESM.docx]

**List of PREDIMED-Plus study investigators**

**Centers in which participants underwent DXA measurements:**

**Rovira i Virgili University, Department of Biochemistry and Biotechnology, Human Nutrition Unit, University Hospital of Sant Joan de Reus, Pere Virgili Institute for Health Research, Reus, Spain:** R. Pedret-Llaberia, R. Gonzalez, R. Sagarra-Álamo, F. París-Palleja, J. Balsells, J.M. Roca, T. Basora-Gallisa, J. Vizcaino, P. Llobet-Alpizarte, C. Anguera-Perpiñá, M. Llauradó-Vernet, C. Caballero, M. Garcia-Barco, M.D. Morán-Martínez, J. García-Rosselló, A. del Pozo, C. Poblet-Calaf, P. Arcelin-Zabal, X. Floresví, M. Ciutat-Benet, J.J. Cabré-Vila, F. Dolz-Andrés, M. Soler, M. Gracia-Vidal, J. Vilalta J. Boj Casajuana, M. Ricard, F. Saiz, A. Isach, M. Sanchez-Marin Martinez, E. Granado-Font, C, Lucena-Luque, C. Mestres-Sola, N. Becerra-Tomás, G. Mestres, J. Basora, G. Mena-Sánchez, L. Barrubés-Piñol, M. Gil-Segura, N. Rosique-Esteban, S. Chig, I. Abellán-Cano, V. Ruiz-García, C. Gomez-Martinez, L. Lopez-Gonzalez, A. Salas-Huetos, I. Paz-Graniel, J. Roig-Vallverdú, C. Claudia Miñana-Garcia, L. Sánchez-Niembro, P. Hernandez-Alonso, S. Canudas, A. Díaz-López.

**Department of Preventive Medicine and Public Health, University of Navarra-Navarra Institute for Health Research (IdiSNA), Pamplona, Spain:** M.A. Martínez-González, E. Toledo, M.A. Martínez-González, Z. Vázquez, C. Razquin, M. Bes-Rastrollo, A. Gea, A. Sanchez-Tainta, B. Sanjulián-Aranguren, E. Goñi, L. Goñi, M.J. Cobo, A. Rico-Campa, F.J. Basterra-Gortari, A. Garcia-Arellano, J. Diez-Espino, O. Lecea-Juarez, J. Carlos Cenoz-Osinaga, I. Alvarez-Alvarez, M.C. Sayon-Orea, C.I. Fernandez-Lázaro, L. Ruiz-Estigarribia, J. Bartolome-Resano, A. Sola-Larraza (†), E. Lozano-Oloriz, B. Cano-Valles, S. Eguaras, E. Pascual Roquet-Jalmar, I. Galilea-Zabalza, H. Lancova, R. Ramallal, M.L. Garcia-Perez, V. Estremera-Urabayen, M.J. Ariz-Arnedo, C. Hijos-Larraz, C. Fernandez-Alfaro, B. Iñigo-Martinez, R. Villanueva-Moreno, S. Martin-Almendros, L. Barandiaran-Bengoetxea, C. Fuertes-Goñi, A. Lezaun-Indurain, M.J. Guruchaga-Arcelus, O. Olmedo-Cruz, L. Escriche-Erviti, R. Ansorena-Ros, R. Sanmatin-Zabaleta, J. Apalategi-Lasa, J. Villanueva-Telleria, M.M. Hernández-Espinosa, L. Herrera-Valdez, L. Dorronsoro-Dorronsoro, L. Echeverria-Lizarraga (†), J.A. Cabeza-Beunza, P. Fernández-Urretavizcaya, P. Gascó-García, C. Royo-Jimenez, J. Moran-Pí, F. Salazar-Fernández, F.J. Chasco-Ros, F. Cortés-Ugalde, J.J. Jurio-Burgui, P. Pascual-Pascual, A.I. Rodríguez-Ezpeleta, M. Esparza-Cáceres, C. Arroyo-Azpa, M. Rodríguez-Sanz de Galdeano, T. Forcen-Alonso, M. Armendariz-Marcotegui, A. Brugos-Larumbe, A. Arillo, B. López-Aisa.

**Hospital Son Espases (HUSE) and Institute for Health Research Illes Balears (IdISBa), Palma de Mallorca, Spain:** M. Moñino, A. Colom, M. Morey, M.A. Martín, E. Rayó, J. Llobera, C. Fernández-Palomeque, E. Fortuny, M. Noris, L. López, X. Rosselló, S. Munuera, F. Tomás, F. Fiol, A. Jover, J.M. Janer, C. Vallespir, I. Mattei, N. Feuerbach, M.M. Sureda, S. Vega, L. Quintana, A. Fiol, M. Amador, S. González, J. Coll, A. Moyá, T. Piqué-Sistac, M.D. Sanmartín-Fernández, M.C. Piña-Valls, M.A. Llorente San Martín, J. Pou-Bordoy.

**Department of Nutrition, Food Sciences, and Physiology, Center for Nutrition Research, University of Navarra, Pamplona, Spain:** I. Cantero, C. Cristobo, I. Ibero-Baraibar, M. Zulet, J. Ágreda-Peiró, M.D. Lezáun-Burgui, N. Goñi-Ruiz, R. Bartolomé-Resano, E. Cano-Cáceres, T. Elcarte-López, E. Echarte-Osacain, B. Pérez-Sanz, I. Blanco-Platero, A. Andueza- Azcárate, A. Gimeno-Aznar, E. Ursúa-Sesma, B. Ojeda-Bilbao, J. Martinez-Jarauta, L. Ugalde-Sarasa, B. Rípodas-Echarte, M.V. Güeto-Rubio, C. Napal-Lecumberri, MD Martínez-Mazo, E Arina-Vergara, A. Parra-Osés, F. Artal-Moneva, F. Bárcena-Amigo, F. Calle-Irastoza, J. Abad-Vicente, J.I. Armendáriz-Artola, P. Iñigo-Cibrian, J. Escribano-Jarauta, J. Ulibarri-delportillo, B. Churio-Beraza, Y. Monzón-Martínez, E. Madoz-Zubillaga, C. Arroniz.

**Department of Internal Medicine, Institut d’Investigacions Biomèdiques August Pi i Sunyer (IDIBAPS), Hospital Clínic, University of Barcelona, Barcelona, Spain:** C. Viñas, S. Castro-Barquero, A.M. Ruiz-León, R. Losno, L. Tarés, A. Jordán, R. Soriano, M. Camafort, C. Sierra, E. Sacanella, J. M. Cots, I. Sarroca, M. García, N. Bermúdez, A. Pérez, I. Duaso, A. de la Arada, R. Hernández, C. Simón, M.A. de la Poza, I. Gil, M. Vila, C. Iglesias, N. Assens, M. Amatller, LL. Rams, T. Benet, G. Fernández, J. Teruel, A. Azorin, M. Cubells, D. López, J.M. Llovet, M.L. Gómez, P. Climente, L. de Paula, J. Soto, C. Carbonell, C. Llor, X. Abat, A. Cama, M. Fortuny, C. Domingo, A. I. Liberal, T. Martínez, E. Yañez, M. J. Nieto, A. Pérez, E. Lloret, C. Carrazoni, A. M. Belles, C. Olmos, M. Ramentol, M. J. Capell, R. Casas, I. Giner, A. Muñoz, R. Martín, E. Moron, A. Bonillo, G. Sánchez, C. Calbó, J. Pous, M. Massip, Y. García, M.C. Massagué, R. Ibañez, J. Llaona, T. Vidal, N. Vizcay, E. Segura, C. Galindo, M. Moreno, M. Caubet, J. Altirriba, G. Fluxà, P. Toribio, E. Torrent, J. J. Anton, A. Viaplana, G. Vieytes, N. Duch, A. Pereira, M. A. Moreno, A. Pérez, E. Sant, J. Gené, H. Calvillo, F. Pont, M. Puig, M. Casasayas, A. Garrich, E. Senar, A. Martínez, I. Boix, E. Sequeira, V. Aragunde, S. Riera, M. Salgado, M. Fuentes, E. Martín, A. Ubieto, F. Pallarés, C. Sala, A. Abilla, S. Moreno, E. Mayor, T. Colom, A. Gaspar, A. Gómez, L. Palacios, R. Garrigosa.

**Biomedicine Institute (IBIOMED); University of León, and Primary Health Care Management of León (Sacyl), León, Spain: Biomedicine Institute (IBIOMED); University of León, and Primary Health Care Management of León (Sacyl), León, Spain:** V. Martín, S. Abajo-Olea, L. Álvarez-Álvarez, M. Rubín-García, A. Torres, P. Farias, N. Cubelos, A. Adlbi Sibai, M. Ajenjo, E. Carriedo-Ule, M. Escobar-Fernández, J.I. Ferradal-García, J.P. Fernández-Vázquez, C. González-Quintana, F. González-Rivero, M. Lavinia-Popescu, J.I. López-Gil, J. López de la Iglesia, A. Marcos-Delgado, C. Merino-Acevedo, S. Reguero-Celada, M. Rodríguez-Bul, E. Fernández-Mielgo.

**Department of Endocrinology, IDIBAPS, Hospital Clinic, University of Barcelona, Barcelona, Spain:** A. Altés, I. Vinagre, C. Mestre, J. Viaplana, M. Serra, J. Vera, T. Freitas, E. Ortega, I. Pla.
